# Comparative Transcriptome Analysis of Two Contrasting Soybean Varieties in Response to Aluminum Toxicity

**DOI:** 10.3390/ijms21124316

**Published:** 2020-06-17

**Authors:** Lijuan Zhao, Jingjing Cui, Yuanyuan Cai, Songnan Yang, Juge Liu, Wei Wang, Junyi Gai, Zhubing Hu, Yan Li

**Affiliations:** 1 National Key Laboratory of Crop Genetics and Germplasm Enhancement, National Center for Soybean Improvement, Key Laboratory for Biology and Genetic Improvement of Soybean (General, Ministry of Agriculture), Jiangsu Collaborative Innovation Center for Modern Crop Production, Nanjing Agricultural University, Nanjing 210095, China; zlj1525@126.com (L.Z.); jingjingcui@genovo.org (J.C.); c1205048398@163.com (Y.C.); ysn785620774@126.com (S.Y.); luoyeyanhua@126.com (J.L.); Weiwang1012@126.com (W.W.); sri@njau.edu.cn (J.G.); 2 College of Life Sciences, Nanjing Agricultural University, Nanjing 210095, China; huzhubing@njau.edu.cn

**Keywords:** aluminum, cellulose, gene ontology (GO) enrichment, RNA-seq, soybean

## Abstract

Aluminum (Al) toxicity is a major factor limiting crop productivity on acid soils. Soybean (*Glycine max*) is an important oil crop and there is great variation in Al tolerance in soybean germplasms. However, only a few Al-tolerance genes have been reported in soybean. Therefore, the purpose of this study was to identify candidate Al tolerance genes by comparative transcriptome analysis of two contrasting soybean varieties in response to Al stress. Two soybean varieties, M90-24 (M) and Pella (P), which showed significant difference in Al tolerance, were used for RNA-seq analysis. We identified a total of 354 Al-tolerance related genes, which showed up-regulated expression by Al in the Al-tolerant soybean variety M and higher transcript levels in M than P under Al stress. These genes were enriched in the Gene Ontology (GO) terms of cellular glucan metabolic process and regulation of transcription. Five out of 11 genes in the enriched GO term of cellular glucan metabolic process encode cellulose synthases, and one cellulose synthase gene (*Glyma.02G205800*) was identified as the key hub gene by co-expression network analysis. Furthermore, treatment of soybean roots with a cellulose biosynthesis inhibitor decreased the Al tolerance, indicating an important role of cellulose production in soybean tolerance to Al toxicity. This study provides a list of candidate genes for further investigation on Al tolerance mechanisms in soybean.

## 1. Introduction

Acid soils comprise up to 50% of the potentially arable land in the world [1,2] and about 21% of total arable land in China [3]. In acid soils, aluminum (Al) toxicity has a detrimental effect on crop productivity [4,5], which can cause 30% to 40% of yield loss in rice and other cereals [6]. Al toxicity can damage and restrain the growth of plant roots, and affect the absorption of nutrients and water, leading to the reduction in crop yield [2,7,8]. Al limits the growth of roots by inhibition of cell division, cell elongation or both [8,9,10], and the undeveloped roots under Al toxicity might be due to the structural and functional damage to roots [11,12].

Studies have suggested that the cell wall is the first barrier when plant roots interact with Al in acid soil, and also the major pool of Al in plants. Al-induced inhibition of cell elongation in the hypocotyl of *Abelmoschus esculentus* was mainly due to the Al-binding in epidermis, and 95% of the Al in epidermis was in the cell wall [13]. Another study found that about 85% of the Al was accumulated in the cell wall of roots in *Zea mays* [14]. Many studies also showed that the main Al-binding sites in cell wall are polysaccharides [15,16,17]. The contents of cell wall polysaccharides, including pectin, hemi-cellulose, and cellulose, were increased by Al in the roots of *Cucurbita maxima* Duch. [18]. In addition to the direct Al-binding, the plant cell wall is also associated with ectopic lignin deposition, and activation of jasmonate and ethylene signalling pathways [19,20]. 

Plants have developed several strategies against Al toxicity, which are categorized into two main mechanisms, including internal tolerance and external detoxification [2,12]. With internal tolerance, plants tolerate Al toxicity either through binding Al to pectin and hemi-cellulose in the root cell wall [17,21] or forming Al chelation that occurs inside the cell and then compartmentalized in other organelles like vacuole. For example, in buckwheat (*Fagopyrum esculentum* Moench), internal detoxification of Al is achieved by Al-oxalate and this complex was sequestrated in the vacuoles of the leaves [22,23,24,25]. Two genes from buckwheat, *FeALS1.1* and *FeALS1.2*, which belong to the half-size ABC transporter genes, were found to play a role in the internal detoxification of Al [26]. For external detoxification, plant root apexes exudate organic acids to chelate Al in the rhizosphere [27,28,29]. The first malate transporter gene, *TaALMT1* (encoding an aluminum-activated malate transporter, ALMT), was isolated from the root tips of Al-tolerant wheat (*Triticum aestivum* L.) ET8 [30]. The malate transporter genes from other crops, such as *BnALMT1* and *BnALMT2* in rape (*Brassica napus*), *GmALMT1* in soybean (*G. max*), and *ScALMT1* in rye (*Secale cereale* L.), shared similar functional characteristics with *TaALMT1* [31,32,33,34]. Multidrug and toxic compound extrusion (MATE) proteins facilitate citrate efflux from plant root apices, which can chelate and detoxify Al^3+^ in the apoplast and rhizosphere around root apices [35]. The *SbMATE1* gene was identified to confer Al tolerance in sorghum (*Sorghum bicolor*), which is the second cloned Al-tolerance gene in plants [35]. Subsequently, *MATE* genes were also found in other crops, for example, *AtMATE* in *Arabidopsis*, *OsFRDL4* in rice, and *ZmMATE1* in maize [28,36,37].

Soybean (*Glycine max* (L.) Merr.) is one of the most important legume crops in the world, which provides oil and proteins for human food and feed [38]. Soybean is a moderately Al-tolerant crop [39], and there is a great variation in Al tolerance among different soybean varieties. Previous genetic [40] and physiological [41] studies showed that Al tolerance in soybean is a complex trait. However, there is limited information on Al tolerance genes in soybean. In addition to the known Al tolerance genes encoding ABC transporter, ALMT, and MATE [26,28,30,36,37], more genes related to Al tolerance need to be explored. In soybean, research on mapping the quantitative trait loci (QTL) underlying Al tolerance and identifying the Al-responsive genes by microarray analysis or cDNA-RAPD has been reported [42,43,44]. To our knowledge, there have been no reports on comparative transcriptomic analysis of soybean response to Al toxicity, which have been performed in other crops [45,46]. Combination of comparative analysis of gene expression profiles and phenotypic difference between Al-tolerant and sensitive soybean varieties could help us to identify candidate genes for Al tolerance. Therefore, in this study, we performed a comparative transcriptome analysis of two contrasting soybean varieties, M90-24 (Al-tolerant, M) and Pella (Al-sensitive, P), under Al stress and control condition, to identify candidate Al tolerance genes and the possible molecular basis of Al tolerance in soybean.

## 2. Results

### 2.1. Relative Root Growth of Two Soybean Varieties under Al Stress

**Figure 1:**
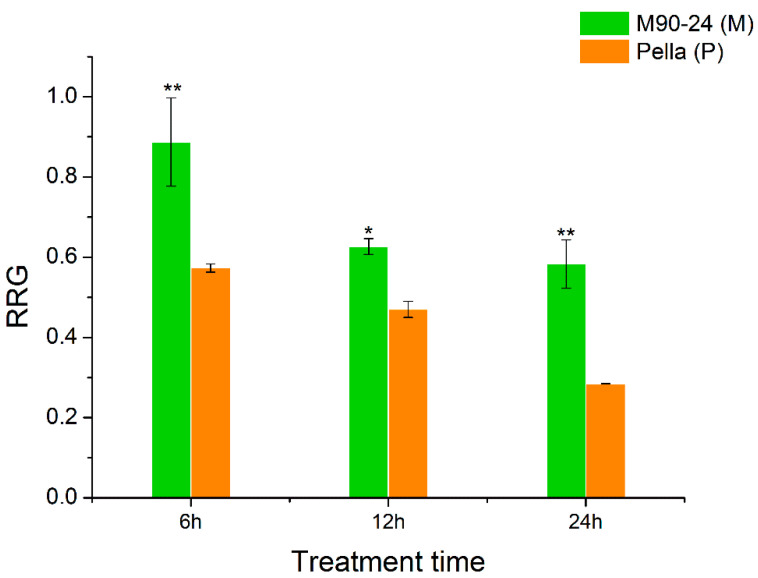
The relative root growth (RRG) of two soybean varieties (primary roots) at 6, 12 and 24 h of Al stress. The control and Al treatment contain 0 and 25 μM AlCl_3_, pH = 4.3, respectively. Data represent mean ± standard error from three biological replications; * and ** indicate significant difference in RRG between two soybean varieties at the 0.05 and 0.01 level, respectively.

Two soybean varieties, M90-24 (M) and Pella (P), were chosen for this study due to their difference in Al tolerance, as shown by their relative root growth (RRG) under Al stress compared with control conditions. The RRG of M was significantly greater than P after 6, 12 and 24 h of 25 μM AlCl_3_ stress (Figure 1). The root growth of P was severely inhibited (RRG = 0.30) after 24 h of Al stress, and therefore we chose 6 and 12 h as the time points to perform the comparative transcriptome analysis using the roots of these two soybean varieties under Al stress and control by RNA-seq. 

### 2.2. RNA-seq Data Quality, Assembly and Annotation

A total of 83.40 GB raw reads and 79.20 GB high-quality clean data were obtained for 16 samples, with the average of 4.89 GB clean data per sample. On average, 95.27% clean reads had Phred-like quality scores at the Q20 level (an error probability of 1%), and 91.71% at the Q30 level (an error probability of 0.1%). The average GC contents were about 45.40%. After assembly, the 16 sets of clean reads were mapped to the soybean reference genome (Wm82.a2.v1). Among the 16 samples, 91.07% to 94.22% of the clean reads were mapped to the soybean genome, and the uniquely mapped percentage ranged from 84.82% to 92.95% (Tables S1 and S2). The gene coverage (genes with FPKM ≥ 0.01) started to show saturation when approximately more than 5 million clean reads were obtained by RNA-seq (Figure S1A), while the average number of clean reads for our 16 samples was 17.43 million, which exceeded the saturation threshold. The sequencing reads were uniformly distributed in the relative position (5′ to 3′) in genes (Figure S1B). These analyses indicate that the quality of our RNA-seq data was high and the sequencing depth was sufficient for further analysis.

### 2.3. Differentially Expressed Genes (DEGs) in Soybean Roots in Response to Al Stress

**Figure 2:**
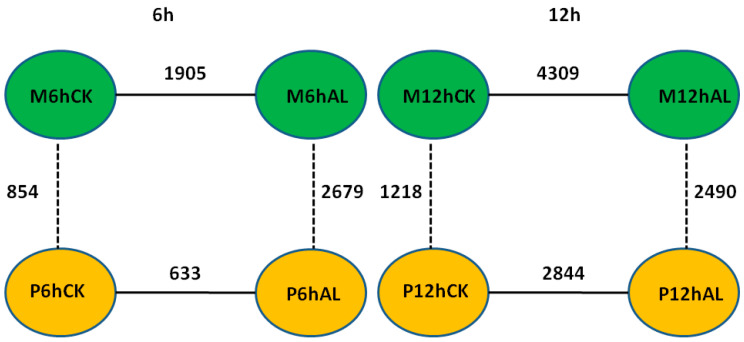
Diagram of the differentially expressed genes (DEGs) identified from different comparisons between samples from the two soybean varieties under Al stress (25 μM AlCl_3_, pH = 4.3) and control (0 μM AlCl_3_, pH = 4.3) at 6 and 12 h.

By using the criteria of false discovery rate (*FDR*) < 0.05 and |log_2_ (fold change)| > 1, the differentially expressed genes (DEGs) between Al stress and control at 6 and 12 h in the roots of two soybean varieties were identified, respectively. There were 1905 and 4309 DEGs between Al stress and control at 6 and 12 h in M, respectively, while there were 633 and 2844 DEGs between Al stress and control at 6 and 12 h in P, respectively (Figure 2). In the Al-tolerant soybean variety M, there were 633 up-regulated and 1272 down-regulated DEGs between Al stress and control at 6 h, respectively, while there were 3176 up-regulated and 1133 down-regulated DEGs between Al stress and control at 12 h, respectively (Table S3). In the Al-sensitive soybean variety P, there were only 169 up-regulated and 464 down-regulated DEGs between Al stress and control at 6 h, respectively, and 2189 up-regulated and 655 down-regulated DEGs between Al stress and control at 12 h, respectively (Table S3). Fifteen and 21 pathways were enriched in up- or down-regulated genes in M, respectively, while 14 and 16 pathways were enriched in up- or down-regulated genes in P, respectively (Table S4). Arginine and proline metabolism (ko00330) as well as photosynthesis (ko00195) were only enriched in the up-regulated genes in the Al tolerant soybean variety M (Table S4). Circadian rhythm-plant (ko04712), flavonoid biosynthesis (ko00941), nitrogen metabolism (ko00910), phenylalanine metabolism (ko00360), plant-pathogen interaction (ko04626), and sulfur metabolism (ko00920) were enriched only in up-regulated genes in both soybean varieties. Six pathways including amino sugar and nucleotide sugar metabolism (ko00520), diterpenoid biosynthesis (ko00904), DNA replication (ko03030), mismatch repair (ko03430), pentose and glucuronate interconversions (ko00040), as well as valine, leucine and isoleucine degradation (ko00280), were enriched only in down-regulated genes in both soybean varieties. Phenylalanine biosynthesis (ko00940) was enriched in both up- and down- regulated genes in both soybean varieties (Table S4).

### 2.4. DEGs between Two Soybean Varieties

The DEGs between two soybean varieties, M and P, were also identified using the criteria of *FDR* < 0.05 and |log_2_ (fold change)| > 1. Under control (CK) conditions, there were 854 and 1218 DEGs between two soybean varieties at 6 and 12 h, respectively, while 2679 and 2490 DEGs were found between two varieties under Al stress at 6 and 12 h, respectively (Figure 2). These results show that at the same time point, there were more DEGs between two soybean varieties under Al stress than control condition, which is likely due to the changes in gene expression upon Al stress. Under control conditions, there were 573 higher-expression and 281 lower-expression genes in M than P at 6 h, respectively, while 749 higher-expression and 469 lower-expression genes in M than P at 12 h, respectively (Table S5). Under Al stress, there were 1574 higher-expression and 1105 lower-expression genes in M than P at 6 h, respectively, while there were 1687 higher-expression and 803 lower-expression genes in M than P at 12 h, respectively (Table S5).

### 2.5. Identification of Candidate Genes Related to Al Tolerance in Soybean 

**Figure 3:**
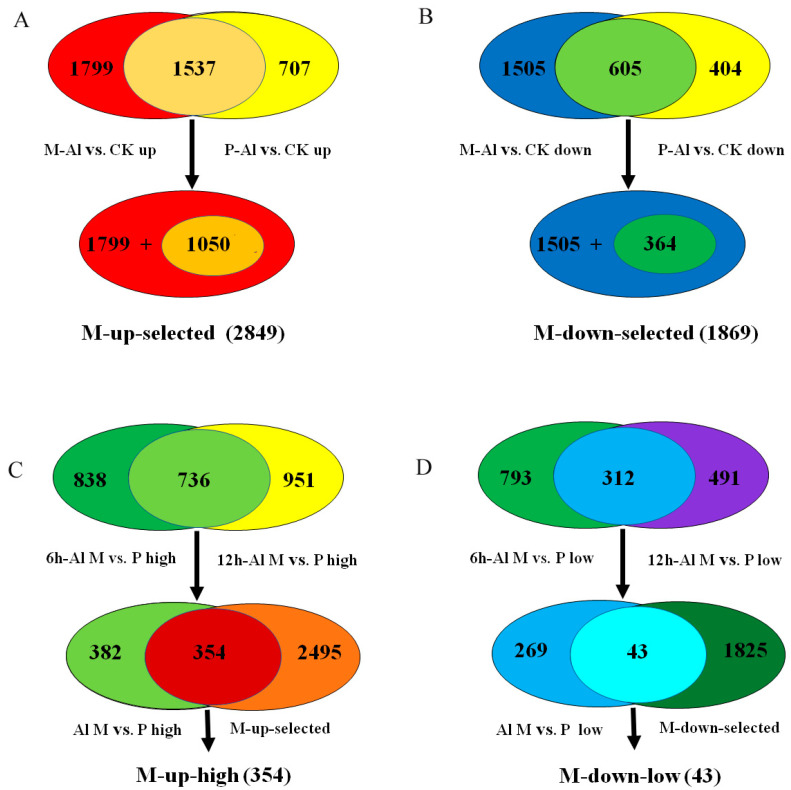
The selection procedure for Al-tolerance related genes in the lists of M-up-selected, M-down-selected, M-up-high and M-down-low. (**A**) The M-up-selected list includes 2849 Al-induced genes specific to M (1799) or with more up-regulation in M than P (1050). (**B**) The M-down-selected list includes 1869 Al-repressed genes specific to M (1505) or with more down-regulation in M than P (364). (**C**) The M-up-high list includes 354 genes with higher relative expression levels in M than P under Al treatment and more up-regulation by Al in M than P. (**D**) The M-down-low list includes 43 genes with lower relative expression levels in M than P under Al treatment and more down-regulation by Al in M than P.

In detail, 3336 and 2244 genes were up-regulated by Al stress in M and P, respectively, among which 1799 Al-up-regulated genes were specific to M and 1537 Al-up-regulated genes overlapped between M and P (Figure 3A). Among the 1537 overlapped Al-up-regulated genes, 1050 genes were up-regulated more in M than P. Together, 2849 Al-up-regulated genes, including 1799 specific to M and 1050 genes overlapped between M and P but with more fold change in M than P, were designated as M-up-selected (Figure 3A, Table S6). Similarly, 1869 Al-down-regulated genes, including 1505 specific to M and 364 overlapped between M and P genes with more down-regulation in M than P, were designated as M-down-selected (Figure 3B, Table S7).

In order to identify the most possible Al-responsive candidate genes for soybean tolerance to Al toxicity, the genes in M-up-selected that also showed higher expression levels in M than P under Al stress at both time points were further selected (354 genes) and designated as M-up-high (Figure 3C, Table S8), and the genes in M-down-selected that showed lower expression levels in M than P under Al stress at both time points (43 genes) were designated as M-down-low (Figure 3D, Table S9). 

### 2.6. Gene Ontology (GO) Enrichment of Candidate Al Tolerance Genes in Soybean

**Figure 4:**
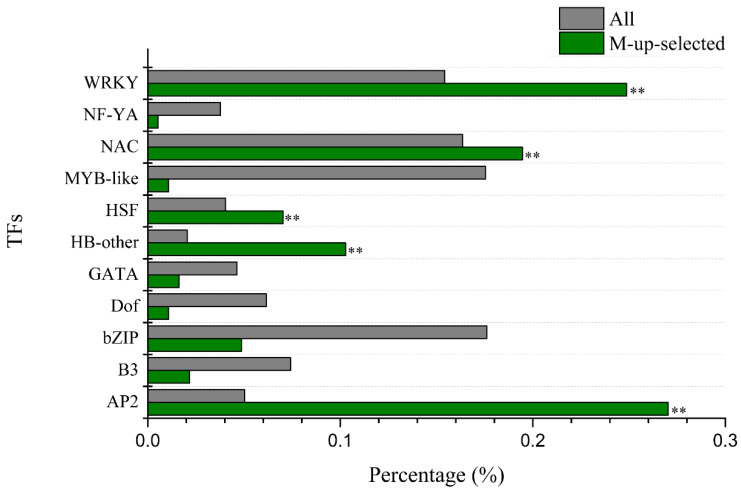
Enrichment of the transcription factor (TF) families among the M-up-selected genes. The percentage of TF families in M-up-selected genes and all TFs in soybean genome was shown as green bars and grey bars, respectively. ** indicate significant enrichment at 0.01 level.

**Figure 5:**
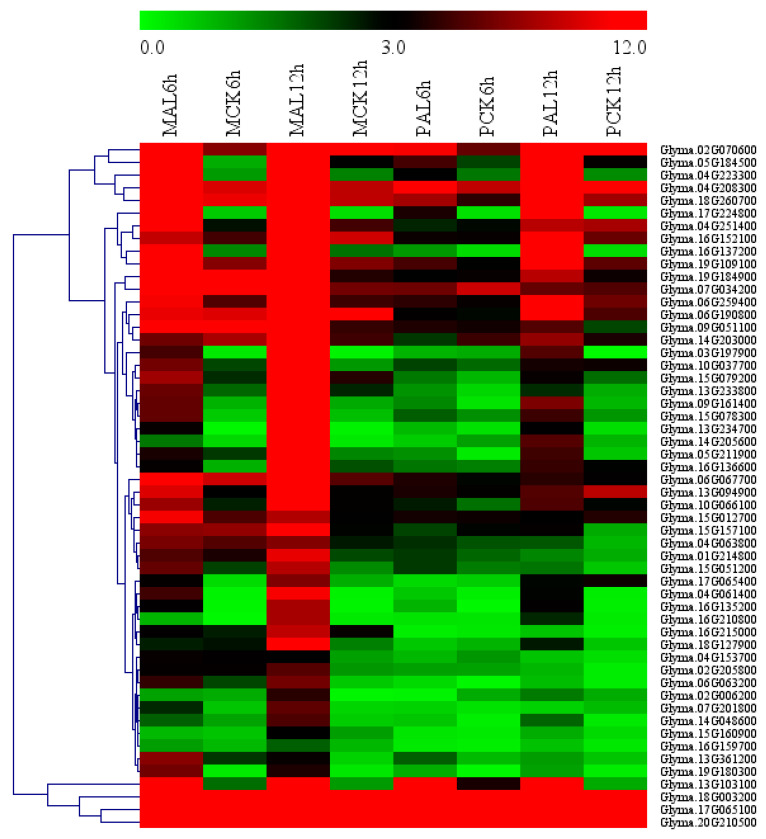
Heatmap of the genes in the enriched GO terms of cellular glucan metabolic process (0006073) and regulation of cellular process (GO:0050794) in M-up-selected list. Heatmap was plotted using Mev4.9 software. Hierarchical clustering of genes was done by complete method with Euclidean distance. The gene expression levels were transformed by log_2_ (FPKM+1) and the values were centered and scaled in row direction. X-axis, samples; Y-axis, gene names.

We used agriGo (*p* < 0.01, *FDR* < 0.05) to perform GO enrichment analysis of candidate Al tolerance genes in soybean variety M. Genes in M-up-selected are significantly enriched in the biological processes (Figure S2) such as DNA-dependent regulation of transcription (GO:0006355, *FDR* = 1.93 × 10^−16^) and protein amino acid phosphorylation (GO:0006468, *FDR* = 1.25 × 10^−8^). There are 207 genes in the GO term of DNA-dependent regulation of transcription, which are enriched in AP2, HB-other, HSF, NAC and WRKY transcription factors (Figure 4). While the M-down-selected genes are significantly enriched in the biological processes (Figure S3) such as DNA replication (GO:0006260, *FDR* = 1.59 × 10^−7^) and oxidation reduction (GO:0055114, *FDR* = 1.59 × 10^−7^), the genes in M-up-high are significantly enriched in the GO terms (Figure S4) related to cellular glucan metabolic process (GO:0006073, *FDR* = 3.76 × 10^−6^) and regulation of cellular process (GO:0050794, *FDR* = 3.88 × 10^−5^). No significantly enriched GO terms were found in M-low-down since there are only 43 DEGs in it. There are 11 genes (Table S10) in the enriched GO term of cellular glucan metabolic process (GO:0006073), including five genes encoding cellulose synthase and six genes encoding xyloglucan endotransglycosylase (XET). There are 43 genes in the enriched GO term of regulation of cellular process (GO:0050794), which includes 30 genes related to DNA-dependent regulation of transcription (GO:0006355, *FDR* = 2.48 × 10^−4^) such as the AP2 domain, AUX/IAA family, NAM, and WRKY DNA binding proteins (Table S11). These 11 and 43 genes showed up-regulated expression by Al in the Al-tolerant soybean variety M and higher transcript levels in M than P under Al stress (Figure 5), as seen in the other DEGs in M-up-high (Table S8). 

### 2.7. Identification of Hub Genes through Co-Expression Network

**Figure 6:**
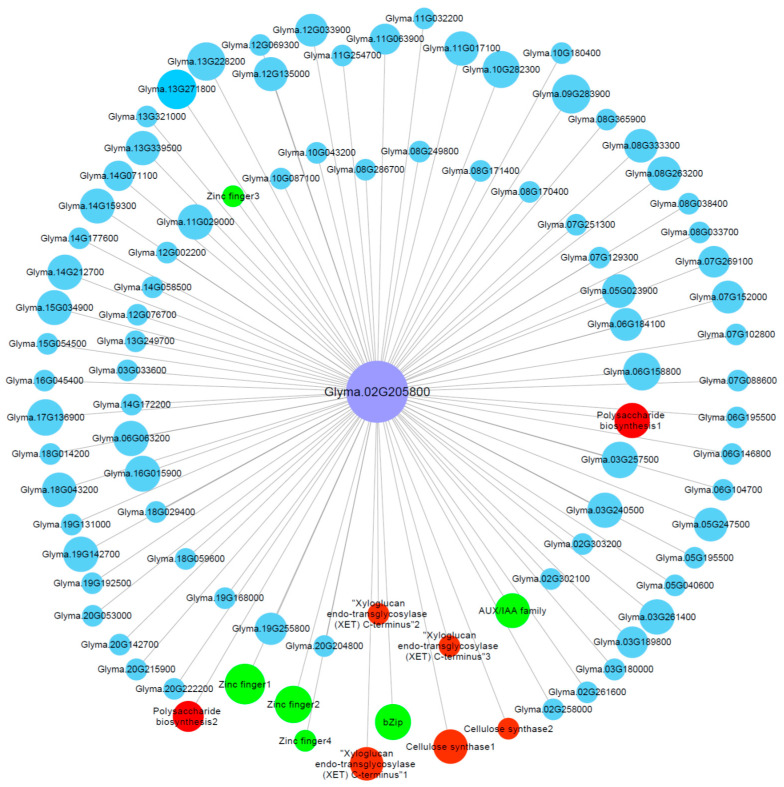
Gene co-expression network for *Glyma.02G205800* (encoding cellulose synthase) in the green module. Only the genes (nodes) with the module membership value greater than 0.3 are shown. The nodes in red represent cell wall related genes, while the nodes in green represent known Al-tolerance related genes.

The co-expression network of the genes in the M-up-selected list (Table S6) was analyzed and a total of six modules were identified (Figure S5). Among these modules, the green module (containing 267 genes) showed a significant (*p* = 7 × 10^−5^) positive correlation (*r* = 0.97) with RRG. To screen the key genes from the green module, we further identified the hub genes via co-expression network in green module. One hub gene, *Glyma.02G205800*, has the highest module membership value, which encodes a cellulose synthase. We then constructed the visual co-expression network of this hub gene (Figure 6) and found that its co-expressed genes include genes encoding AUX/IAA transcription factor (*Glyma.20G210500*), bZip transcription factor (*Glyma.05G182500*), cell wall related proteins such as cellulose synthases (*Glyma.04G063800* and *Glyma.04G153700*), XET (*Glyma.17G064900*, *Glyma.18G003200* and *Glyma.19G184900*), polysaccharide biosynthesis-related proteins (*Glyma.16G001600* and *Glyma.17G169200*), zinc finger proteins (*Glyma.06G076600*, *Glyma.14G177900*, *Glyma.15G072700* and *Glyma.18G074600*), and others. Zinc finger proteins, bZip transcription factors, cell wall-related proteins and AUX/IAA transcription factors have been shown to be associated with Al tolerance [47,48,49,50], which indicates that the hub gene *Glyma.02G205800*, together with its co-expressed genes, might play important roles in soybean tolerance to Al toxicity.

### 2.8. Validation of RNA-seq Data by Quantitative Real Time PCR (qRT-PCR) Analysis 

**Figure 7:**
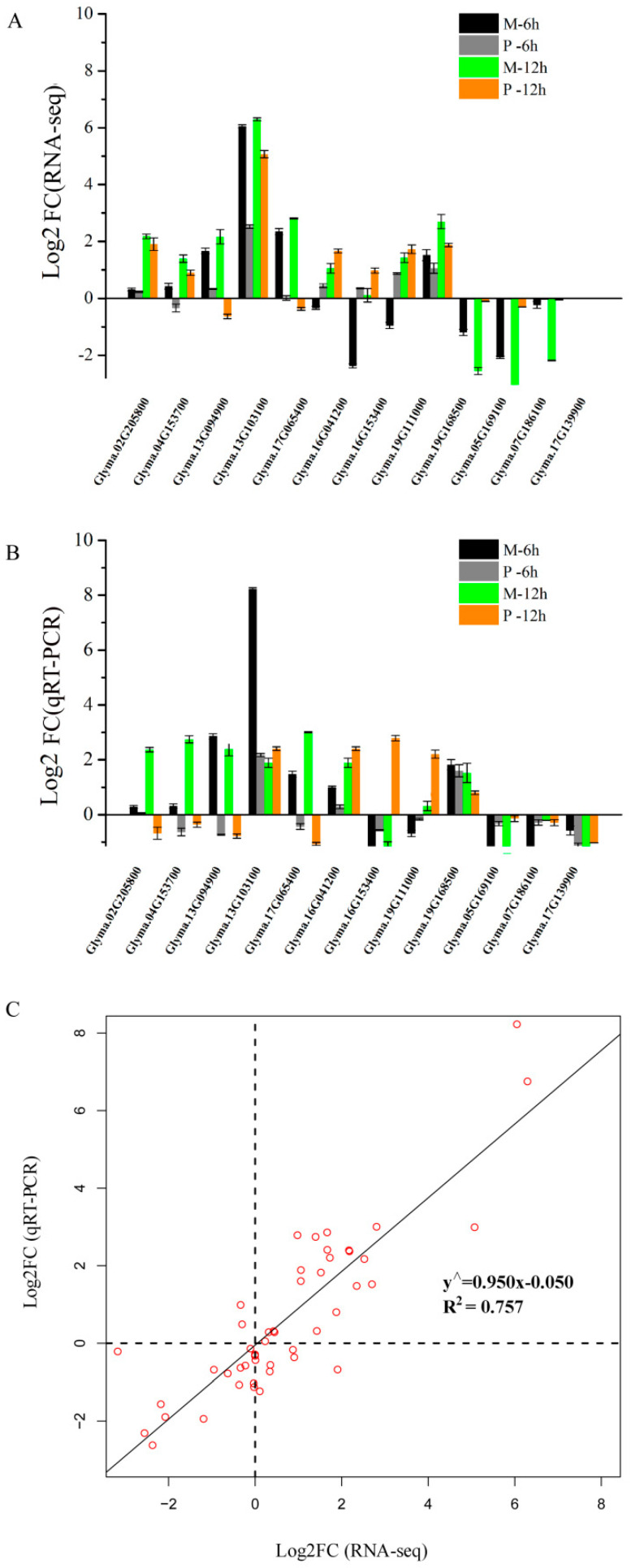
Comparison of the relative expression levels of 12 genes by RNA-seq and qRT-PCR analysis. (**A**) Relative expression of 12 genes at 6 and 12 h after 25 µM AlCl_3_ (pH 4.3) stress compared with control (0 µM AlCl_3_, pH 4.3) by RNA-seq. (**B**) Relative expression of 12 genes at 6 and 12 h after 25 µM AlCl_3_ (pH 4.3) stress compared with control (0 µM AlCl_3_, pH 4.3) by qRT-PCR. (**C**) The correlation analysis between qRT-PCR and RNA-seq using Log_2_ FC. FC represents fold change. *Glyma.02G205800* and *Glyma.04G153700* encode cellulose synthases; *Glyma.13G094900*, *Glyma.13G103100* and *Glyma.17G065400* encode xyloglucan endo-transglycosylases (XET); *Glyma.16G041200*, *Glyma.16G041200*, and *Glyma.19G111000* encode glutamate dehydrogenases; *Glyma.19G168500* encodes an auxin-induced protein (AUX/IAA family); *Glyma.05G169100* belongs to the cupin family with nutrient reservoir activity; *Glyma.07G186100* encodes a branched chain aminotransferase; *Glyma.17G139900* encodes a seed storage protein.

The relative gene expression levels from RNA-seq were verified by qRT-PCR using 12 DEGs (Table S12), including six genes from M-up-high (two cellulose synthase genes, three XET genes, and one AUX/IAA family gene), three genes from enriched KEGG pathways and three genes from M-down-low. The expression profiles of these 12 genes showed similar patterns by RNA-seq and qRT-PCR (Figure 7A,B), and a high consistency was obtained between two methods (Figure 7C). 

### 2.9. Role of Cellulose Synthase Genes in Soybean Tolerance to Al Toxicity 

**Figure 8:**
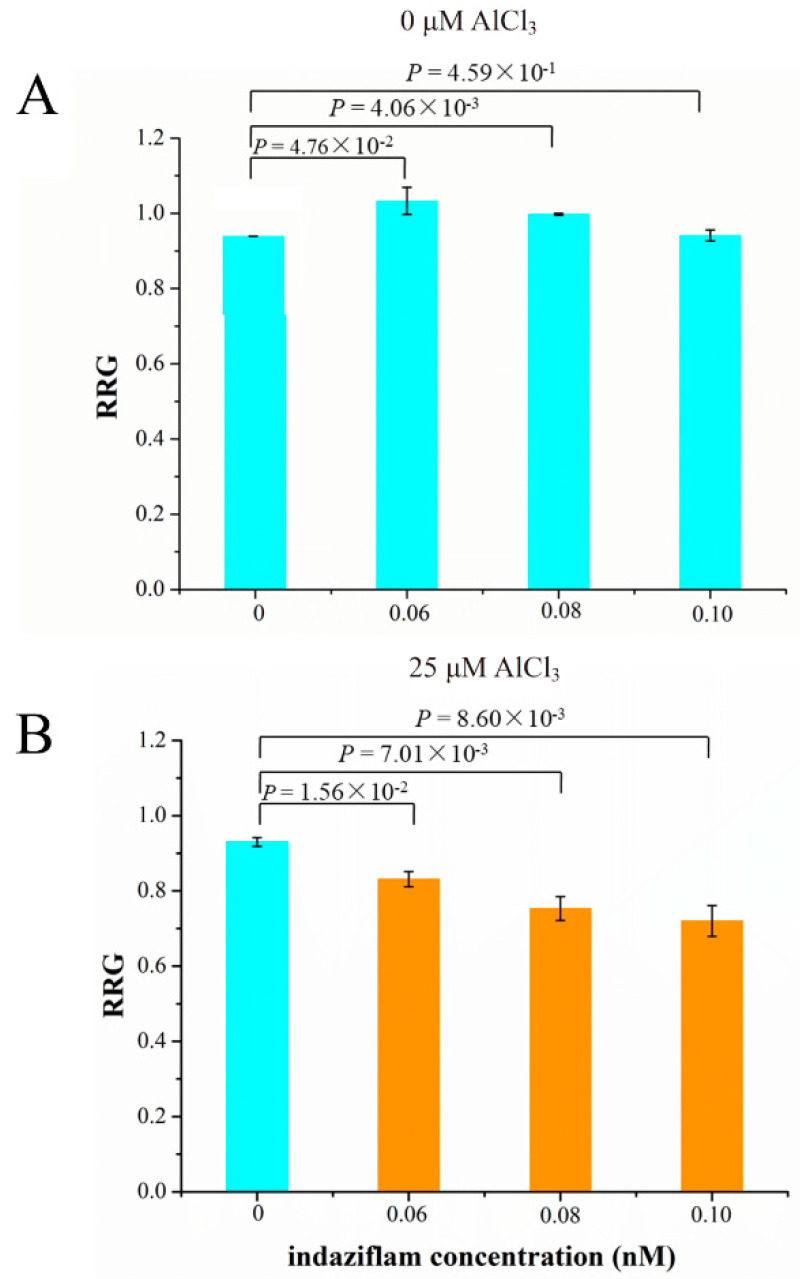
The effect of cellulose biosynthesis inhibitor (indaziflam) on the relative root growth (RRG) of soybean primary roots under 0 (**A**) or 25 μM AlCl_3_ (**B**). *P* values above the bars indicate the significant levels by Student’s *t*-tests.

The relative expression levels of five cellulose synthase genes were up-regulated by Al stress and were higher in M than P (Table S10). Therefore, we investigated the relationship between cellulose production and Al tolerance. We used the cellulose biosynthesis inhibitor (CBI), indaziflam, to test if inhibition of cellulose production leads to decreased Al tolerance (RRG) in soybean. Indaziflam showed strong CBI activity in the dicotyledonous plant (*Arabidopsis thaliana* L.), which could inhibit the production of cellulose less than 1 h [51]. We used three concentrations of indaziflam, 0.06, 0.08 and 0.10 nM, to test their effect on the RRG of soybean under Al stress and control conditions. Under control condition (0 μM AlCl_3_), the RRG was simulated at 0.06 and 0.08 nM indaziflam, but was not significantly affected at 0.10 nM comparing with 0 nM indaziflam (Figure 8A). Under 25 μM AlCl_3_ stress (Figure 8B), the RRG decreased significantly by 0.06, 0.08 and 0.10 nM indaziflam in a dosage-dependent manner, comparing with 0 nM indaziflam, indicating that indaziflam further reduced RRG under Al stress, which coincides with the fact that the expression levels of the five cellulose synthase genes in Al-sensitive variety P are lower than the Al-tolerant variety M. These results therefore support our hypothesis that decreased cellulose production leads to more Al induced inhibition of root growth (more sensitive to Al toxicity) in soybean.

## 3. Discussion

The most significant effect of Al toxicity is the inhibition of root elongation, and the root apex is the most obvious area of Al damage [21,52]. In our study, soybean variety M had the average RRG of 67% and P had the average RRG of 30% after 24 h of 25 μM AlCl_3_ treatment (Figure 1). In order to identify Al tolerance candidate genes and the possible molecular basis of Al tolerance in soybean, we performed a comparative transcriptome analysis of the root tips under Al stress and control between M (Al-tolerant) and P (Al-sensitive). We proposed that the following factors might be related to Al tolerance in soybean.

Cellulose, as an important component of the plant cell wall, plays a critical role against abiotic stresses, including Al toxicity. Al stress disturbs the cellulose synthase conformation [53], which results in lowering the cellulose production, indicating that Al induced growth inhibition might be partially dependent on the decreased cellulose. Indaziflam, a cellulose biosynthesis inhibitor (CBI), can deplete the cellulose synthase complexes on the plasma membrane, finally blocking the cellulose production [51]. CBI-treated plants exhibited severe growth inhibition, accompanied by the swelling of root tip cells [51]. In this study, we found that indaziflam treatment led to more Al-induced inhibition of root growth (more Al-sensitive) in soybean. These results suggest that cellulose production plays an important role in soybean tolerance to Al toxicity. In recent years, it has been found that the content of cellulose in plant roots changes under environmental stresses, such as temperature [54,55] and salt stress [56,57]. Teraoka et al. [53] found that under Al stress, the cell wall cellulose content in the wheat root tip cells of an Al-sensitive cultivar decreased more than in those of the Al-tolerant cultivar, accompanied by apical swelling and inhibition of root growth. The changes in the cellulose content of soybean roots under Al stress have not been reported, and we will continue to conduct in-depth studies on the role of cellulose synthase and cellulose content in soybean tolerance to Al stress in the future, and the changes in root cell expansion under Al stress will also be observed by microscopy. 

In addition to cellulose synthase, there were six XET genes in the enriched GO term of cellular glucan metabolic process (GO:0006073) among the M-up-high list, which showed up-regulated expression by Al in M and higher transcript levels in M than P under Al stress. XET is involved in cell wall extension and participates in cell expansion [58]. Under Al stress, Al binds to various cell wall components, which influences the symplastic cell functions of the cell wall [21]. In this study, compared with Al-sensitive soybean variety P, higher XET gene expression in Al-tolerant M may reduce the Al-toxicity caused by reduced elasticity and plasticity of cell wall. 

Because soybean variety M is more tolerant to Al stress than P (Figure 1), the Al-responsive DEGs that were specific to M or genes that were more differentially expressed (up or down regulated) in M than P would be the first group of candidates related to Al tolerance (Figure 3A,B). These candidate genes could be further narrowed down to the genes that also showed higher expression levels in M than P for up-regulated genes or lower expression levels in M than P for down-regulated genes under Al stress at both time points (Figure 3C,D). In the M-up-selected genes that showed either specificity to M or more up-regulation in M than P, the GO:0006355 term (DNA-dependent regulation of transcription) was significantly enriched. There were 207 DEGs in this term, including 185 transcription factors, which were divided into 11 groups (AP2, B3, bZIP, Dof, GATA, HB-other, HSF, MYB-like, NAC, NF-YA, and WRKY). Among these, AP2, HB-other, HSF, NAC and WRKY transcription factors were significantly enriched (Figure 4).

The most abundant transcription factor family in the M-up-selected gene list was AP2, with a total number of 50 genes. The expression levels of 45 AP2 genes were up-regulated more in the Al-tolerant variety M than that in sensitive variety P at 12 h after Al treatment, and the fold change of the remaining five AP2 genes was higher in M than P after 6 h Al treatment. Although there is no direct evidence that AP2 transcription factor is associated with Al tolerance, it has been found that AP2 / ERF transcription factors are involved in various signaling pathways such as abscisic acid (ABA), salicylic acid (SA), jasmonic acid, and ethylene [59,60,61,62]. ABA and SA have been shown to play important roles in Al tolerance. It has been demonstrated that elevated levels of ABA can promote Al detoxification in buckwheat [63], while exogenous SA can improve the tolerance to Al stress [60] and reduce the oxidative stress caused by Al toxicity [64]. 

There were 46 genes encoding WRKY transcription factors in the M-up-selected gene list, and 41 of them were up-regulated more in the Al-tolerant variety M than that in the sensitive variety P at 12 h of Al stress, and the fold change of remaining five genes was higher in M than that in P at 6 h of Al treatment. It has been found that *WRKY69* was induced by Al in maize and expressed higher in Al tolerant line than Al-sensitive line [65]. Another gene *AtWRKY33* was also up-regulated by Al in *Arabidopsis* [66]. The role of WRKY transcription factors in soybean tolerance to Al toxicity needs further investigation.

Fifteen genes encoding the AUX/IAA family were found in the M-up-selected gene list. AUX/IAA proteins participate in the auxin signaling pathway and play important roles in plant development and stress tolerance [49,50]. It has been shown that Al induced auxin accumulation in soybean roots, and IAA enhanced the expression of *GmMATE* under Al stress [64]. In addition, Al-induced endogenous IAA accumulation significantly correlated with malate exudation, and exogenous treatment with 10 µM IAA or 30 µM N-1-naphthyl-phthalamc acid (NPA, its efflux transport inhibitor) enhanced or decreased malate efflux in wheat roots, thus demonstrating a possible role of IAA in alleviating Al toxicity [67].

In addition to the above genes in the enriched GO terms, four genes encoding ABC transporters (*Glyma.06G0191400*, *Glyma.08G106900*, *Glyma.08G342600*, *Glyma.16G078100*) and three MATE genes (*Glyma.02G090100*, *Glyma.10G267700*, *Glyma.16G157300*) were also found in the M-up-selected list (Table S6), which showed more up-regulation in the Al-tolerant M than Al-sensitive P. Studies have shown that ABC transporter and *MATE* genes play important roles in plant tolerance to Al toxicity. For example, two ABC transporter genes (*STAR1* and *STAR2*) were induced by Al and required for Al detoxification in rice [68]. The *SbMATE1* gene in sorghum was induced by Al and enhanced root citrate exudation [35]. Therefore, the four ABC transporter genes and three *MATE* genes in the M-up-selected list might also play important roles in soybean tolerance to Al toxicity. The *GmALMT1* gene has been found to be associated with Al tolerance in soybean [33]. However, *ALMT* genes did not appear in our M-up-selected list, which suggests that different soybean varieties might have different genes and mechanisms to confer Al tolerance.

Previously, it has been reported that ABC transporter, ALMT, cellulose synthase, MATE, zinc finger proteins might be associated with Al-tolerance in *Arabidopsis*, maize, buckwheat, and wheat [26,28,30,36,69]. In soybean, several Al-tolerance related genes have been reported, such as *ALMT*, *MATE* and *STOP1* [33,70,71]. However, so far, there have been no reports on the role of cellulose genes in soybean Al tolerance, and therefore our study will shed light on the genes and mechanisms underlying soybean Al tolerance.

## 4. Materials and Methods

### 4.1. Plant Growth Conditions and Stress Treatment 

Seeds of two soybean varieties, M90-24 (M) and Pella (P), were obtained from the National Center for Soybean Improvement, Nanjing, China. Soybean seeds were washed thoroughly with tap water for 1 min, then 3 times with distilled water, and germinated in clean sand for three days at 26 °C in the dark. After germination, uniform seedlings were selected and transferred to 500 mL plastic beakers (six seedlings per beaker) filled with 0.5 mM CaCl_2_ solution (pH 4.3) for 24 h. Then the seedlings were subjected to 0.5 mM CaCl_2_ solution (pH 4.3) containing 0 (control) or 25 µM AlCl_3_ for 6, 12 or 24 h. There were four beakers for each soybean variety, two for control (0 µM AlCl_3_, pH4.3) and the other two for Al treatment (25 µM AlCl_3_, pH4.3). Plants were grown in a well-controlled growth room with the temperature regime of 28/22 °C (day/night), photoperiod of 14/10 h (light/dark), light intensity of 45,000 lx and relative humidity of 30–40%. Three independent experiments were performed, and there were two replications within each experiment, with six seedlings per treatment per replication.

### 4.2. Root Length Measurement and Relative Root Growth

Length of the primary root for each soybean seedling was measured using a ruler at 0, 6, 12 and 24 h of treatment (the roots were immersed in sterile water when measuring to avoid dehydration damage). The Al tolerance can be indicated by the relative root growth (RRG) as described previously [72]. The RRG can be calculated by the following formula:

(1) $$RG=RL_{t}-RL_{0}$$

(2) $$RRG=RG_{Al}⁄RG_{c}$$

In Equation (1), root growth (RG) is the difference between the average root length after 6, 12, or 24 h of treatment (RL_t_) and the average root length before (0 h) treatment (RL_0_). In Equation (2), RRG is the ratio of RG under Al treatment and control. 

### 4.3. Sample Collection 

The root tips were quickly washed with distilled water, and then cut 1 cm from root apex from 6 seedlings (pooled together as one sample) and immediately frozen in liquid nitrogen. The samples were stored at −80 °C before use. 

### 4.4. RNA Isolation and RNA-seq Library Preparation

Total RNA was extracted with TRIzol reagent (Invitrogen, Walthman, Massachusetts, USA) from 1 g of frozen root tips. After DNase I treatment, the quantity and quality of total RNA was assessed by measuring the A_260/280_ ratio and electrophoresis on a 1% formaldehyde-agarose gel. The mRNA was isolated by oligo (dT)-attached magnetic beads and subsequently fragmented using divalent cations under elevated temperature. Sixteen libraries were constructed as described previously [73] using NEB Next Ultra Directional RNA Library Prep Kit for Illumina. All libraries were sequenced by Illumina HiSeq 2500 at Berry Genomics Institute, Beijing, China. The RNA-seq data were submitted to NCBI database with the SRA accession number PRJNA591095. 

### 4.5. RNA-seq Analysis

The FastQC (http://www.bioinformatics.babraham.ac.uk/projects/fastqc/) software package [74] was used for the quality control of raw data, and then SolexaQA_v.2.5 [75] was used to filter the poor-quality bases and trim the reads of which length was shorter than the cut-off value of 25. We discarded the reads containing exceeded N and low-quality bases to get the clean reads for further analysis. The clean reads were then aligned to the soybean reference genome (Wm82a2.v1) using TopHat version 2.0.13 [76]. Then Cufflinks v2.1.1 [77] and Cuffmerge [78] were used to assemble the transcriptomes and quantify the gene expression level (fragments per kilobase per million, FPKM). Next, Cuffdiff [79] was used to find differentially expressed genes (DEGs) for different comparisons. In this study, DEGs were identified with the thresholds of *FDR* < 0.05 and |log_2_ (fold change) | >1. The heat map with clustering analysis was constructed using log_2_ (FPKM + 1) by Mev4.9 software.

### 4.6. Gene Annotation, Classification and Enrichment Analysis

Genes were annotated based on the soybean genome annotation in SoyBase (https://www.soybase.org). All DEGs were compared to Kyoto Encyclopedia of Genes and Genomes (KEGG) for functional classifications [80]. Transcription factors were aligned to the Plant transcription factors Database 3.0 [81] and Protein family (Pfam) database [82] using the hmmscan v3.0 software [83]. Fisher’s exact test (*p* < 0.01) and Benjamini–Kochberg procedure (*FDR* < 0.05) were performed to detect statistical enrichment of transcription factors (TFs) and KEGG pathways, in comparison with the soybean genome as the background. Gene Ontology (GO) enrichment analyses were performed using Singular Enrichment Analysis (SEA) method with *p* < 0.01 and *FDR* < 0.05 by agriGO [84]. 

### 4.7. Gene Co-Expression Network Analysis

The FPKM values of the genes in M-up-selected list were used for gene co-expression network analysis by WGCNA (version 1.49) [85]. The co-expressed gene sets (modules) were detected using the hierarchical clustering method. TOM and DynamicTreeCut functions were used for network construction and consensus module detection. After exploring the soft thresholds, we finally set the power to 7, and minimum module size as 30. The correlation between genes as well as the correlation between RRG and each module was measured by Pearson’s correlation. The expression patterns of the modules were analyzed by STEM [86], and the visual co-expression network was constructed using Cytoscape (Version 3.6.1, The Cytoscape Consoritum, USA) [87].

### 4.8. Quantitative Real-Time PCR 

A total of 12 candidate genes were selected to validate the RNA-seq data by quantitative real time PCR (qRT-PCR). We used 1000 ng of total RNA for synthesizing the first-strand cDNAs. The qRT-PCRs were performed with LightCycler 480 instrument (Roche, Penzberg, Upper Bavaria, Germany), in a final volume of 20 μL reaction solution containing 2 μL of cDNA, 10 μL of 2X SYBR Green qPCR Mix (Takara, Japan) and 200 nM of the forward and reverse primers (Table S12). The thermal cycling conditions were set as follows: 40 cycles of 95 °C denaturation for 5 s, 58 °C for 30 s, and 72 °C for 45 s. The reference gene *GmEF-1*α was used as the internal control [33]. 

### 4.9. Indaziflam Treatment

After germination, three-day-old seedlings were transferred to 500 mL plastic beakers filled with 0.5 mM CaCl_2_ solution (pH 4.3) for 24 h. Then these seedlings were transferred to 0.5 mM CaCl_2_ solution (pH 4.3) solutions containing 0 or 25 µM AlCl_3_ with different concentrations of indaziflam (0, 0.06, 0.08 and 0.10 nM, respectively). The root length was measured and RRG was calculated as described above, except that the RRG is the ratio of RG under indaziflam (0.06, 0.08 and 0.10 nM) treatment and control (0 nM indaziflam). 

### 4.10. Statistical Analysis

The RNA-seq analysis was described as above, and Student’s *t*-tests were performed using Excel.

## 5. Conclusions

Two soybean varieties, M and P, showed significant differences in Al tolerance (RRG) in this study. A total of 1799 genes showed up-regulation by Al stress specific to the Al-tolerant variety M but not the Al-sensitive variety P, and 1050 genes were induced by Al in both varieties but with more fold change in M than P. There were 354 out of these 2849 genes that also showed higher expression levels in M than P under Al stress, which were enriched in the GO terms of cellular glucan metabolic process and regulation of transcription. There are five genes encoding cellulose synthases in the enriched GO term of cellular glucan metabolic process, and the treatment of soybean roots with the cellulose biosynthesis inhibitor resulted in decreased Al tolerance. These results suggest that cellulose production may play an important role in soybean tolerance to Al toxicity. This study also provides a list of candidate genes for further investigation on Al tolerance mechanisms and molecular breeding in soybean.

## Supplementary Materials

The following are available online at https://www.mdpi.com/1422-0067/21/12/4316/s1, Figure S1: Sequencing saturation curves (A) and gene coverage analysis (B) of the 16 RNA-Seq samples; Figure S2: Gene ontology (GO) enrichment analysis of DEGs in M-up-selected; Figure S3: Gene ontology (GO) enrichment analysis of DEGs in M-down-selected; Figure S4: Gene ontology (GO) enrichment analysis of DEGs in M-up-high; Figure S5: Correlation between gene co-expression network modules and RRG; Table S1: Summary of the RNA-seq data in this study; Table S2: Detailed information of the RNA-seq data in this study; Table S3: Differentially Expressed Genes between Al and control (CK) in two soybean varieties of M90-24 (M) and Pella (P), respectively; Table S4: Significantly enriched KEGG Pathways in up- or down-regulated genes in M or P in response to 25 μM AlCl3 (pH = 4.3) stress; Table S5: Differentially Expressed Genes between two soybean varieties of M90-24 (M) and Pella (P) under Al or control (CK) condition; Table S6: M-up-selected genes including the Al-responsive genes that showed specific to M or more up-regulation in M than P; Table S7: M-down-selected genes including the Al-responsive genes that showed specific to M or with more down-regulation in M than P; Table S8: Differently expressed genes in M-up-high list; Table S9: Differently expressed genes in M-down-low list; Table S10: Genes in the GO (0006073) term of cellular glucan metabolic process in M-up-high list; Table S11: Genes in the GO (0050794) term of regulation of cellular process in M-up-high list; Table S12: Primer pairs for qRT-PCR.

## Abbreviations

ABA	abscisic acid
ALMT	aluminum-activated malate transporter
CBI	cellulose biosynthesis inhibitor
DEG	differently expressed gene
FDR	false discovery rate
FPKM	fragments per kilobase per million
GO	Gene Ontology
KEGG	Kyoto Encyclopedia of Genes and Genomes
MATE	multidrug and toxin extrusion
RG	root growth
RRG	relative root growth
RNA-Seq	RNA sequencing
SA	salicylic acid
SEA	singular enrichment analysis
TF	transcription factor
XET	xyloglucan endo-transglycosylase
