# Supplementary material for: Comparative Transcriptome Analysis of Two Contrasting Soybean Varieties in Response to Aluminum Toxicity
Source: Int J Mol Sci. 2020 Jun 17;21(12):4316. doi: 10.3390/ijms21124316 (PMC7352676; doi:10.3390/ijms21124316)
Supplement: Supplementary file 1 [file ijms-21-04316-s001.zip › Supplementary Materials 2020June13/Table S1.docx]

**Supplementary Table S1: Summary of the RNA-seq data in this study**

| **Summary** | **Min** | **Max** | **Total** | **Average** |
| --- | --- | --- | --- | --- |
| Total raw reads | 15,548,716 | 27,630,905 | 316,815,919 | 19,800,995 |
| Total clean reads | 13,637,227 | 24,877,352 | 278,903,397 | 17,431,462 |
| Total clean reads/Total raw reads | 84.26% | 91.25% | 88.03% | 88.03% |
| Total raw bases | 4,047,967,500 | 7,169,827,250 | 83,397,277,500 | 5,212,329,844 |
| Total clean bases | 3,887,179,000 | 6,907,726,250 | 79,203,979,750 | 4,887,715,221 |
| GC content | 44.48% | 47.10% | -- | 45.40% |
| Q20 | 94.20% | 96.48% | -- | 95.27% |
| Q30 | 90.15% | 93.56% | -- | 91.71% |
| Total mapped reads | 12,763,266 | 23,349,883 | 259,182,129 | 16,198,883 |
| Total mapped reads/Total clean reads | 91.07% | 94.22% | -- | 92.92% |
| Multiple mapped reads | 1,169,570 | 2,304,270 | 24,987,556 | 1,561,722 |
| Multiple mapped reads/Total mapped reads | 7.05% | 15.18% | -- | 9.82% |
| Uniquely mapped reads | 10,849,559 | 21,703,969 | 234,194,573 | 14,637,161 |
| Uniquely mapped reads/Total mapped reads | 84.82% | 92.95% | -- | 90.18% |
