# Supplementary material for: Comparative Transcriptome Analysis of Two Contrasting Soybean Varieties in Response to Aluminum Toxicity
Source: Int J Mol Sci. 2020 Jun 17;21(12):4316. doi: 10.3390/ijms21124316 (PMC7352676; doi:10.3390/ijms21124316)
Supplement: Supplementary file 1 [file ijms-21-04316-s001.zip › Supplementary Materials 2020June13/Table S3.docx]

**Table S3.** Differentially Expressed Genes between Al and control (CK) in two soybean varieties of M90-24 (M) and Pella (P), respectively.

| Comparisons | Samples | Up-regulated DEGs | Down-regulated DEGs |
| --- | --- | --- | --- |
| 1. M-6h-Al vs. CK | M6hAl vs. M6hCK | 633 | 1272 |
| 1. M-12h-Al vs. CK | M12hAl vs. M12hCK | 3176 | 1133 |
| (3) P-6h-Al vs. CK | P6hAl vs. P6hCK | 169 | 464 |
| (4) P-12h-Al vs. CK | P12hAl vs. P12hCK | 2189 | 655 |
