# Supplementary material for: Comparative Transcriptome Analysis of Two Contrasting Soybean Varieties in Response to Aluminum Toxicity
Source: Int J Mol Sci. 2020 Jun 17;21(12):4316. doi: 10.3390/ijms21124316 (PMC7352676; doi:10.3390/ijms21124316)
Supplement: Supplementary file 1 [file ijms-21-04316-s001.zip › Supplementary Materials 2020June13/Table S5.docx]

**Table S5.** Differentially Expressed Genes between two soybean varieties of M90-24 (M) and Pella (P) under Al or control (CK) condition.

| Comparisons | Samples | Up-regulated DEGs | Down-regulated DEGs |
| --- | --- | --- | --- |
| (1) 6h-CK M vs. P | M6hCK vs. P6hCK | 573 | 281 |
| (2) 6h-Al M vs. P | M6hAl vs. P6hAl | 1574 | 1105 |
| (3) 12h-CK M vs. P | M12hCK vs. P12hCK | 749 | 469 |
| (4) 12h-Al M vs. P | M12hAl vs. P12hAl | 1687 | 803 |
