# Supplementary figures and images for: Comparative Transcriptome Analysis of Two Contrasting Soybean Varieties in Response to Aluminum Toxicity
Source: Int J Mol Sci. 2020 Jun 17;21(12):4316. doi: 10.3390/ijms21124316 (PMC7352676; doi:10.3390/ijms21124316)

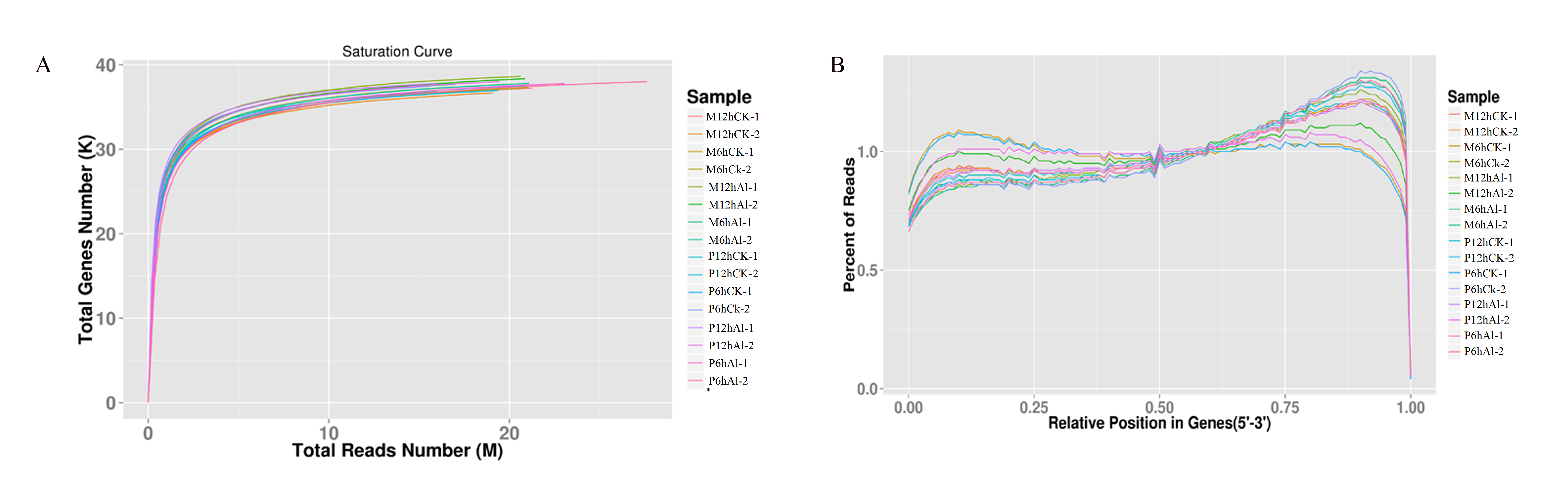

Supplement: Supplementary file 1 [file ijms-21-04316-s001.zip › Supplementary Materials 2020June13/Figure S1.tif]

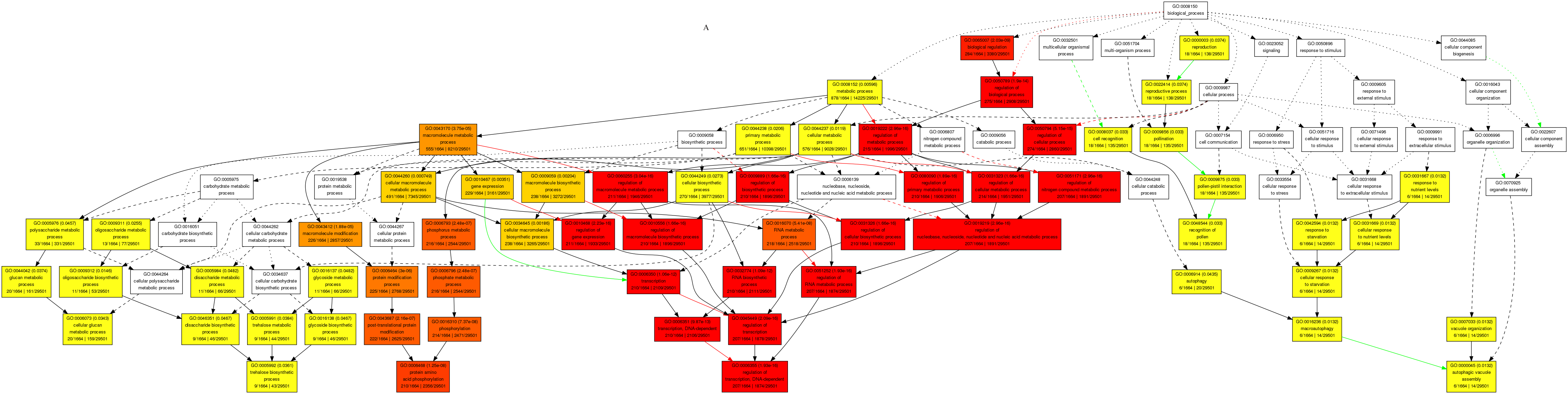

Supplement: Supplementary file 1 [file ijms-21-04316-s001.zip › Supplementary Materials 2020June13/Figure S2.tif]

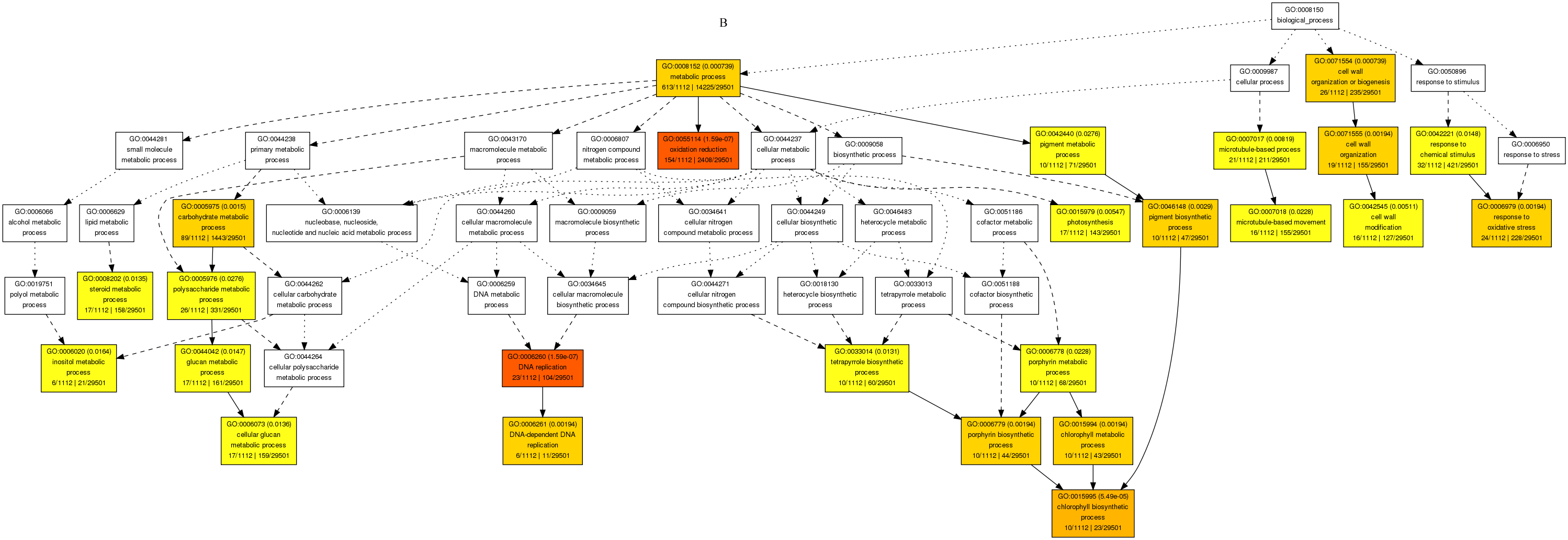

Supplement: Supplementary file 1 [file ijms-21-04316-s001.zip › Supplementary Materials 2020June13/Figure S3.tif]

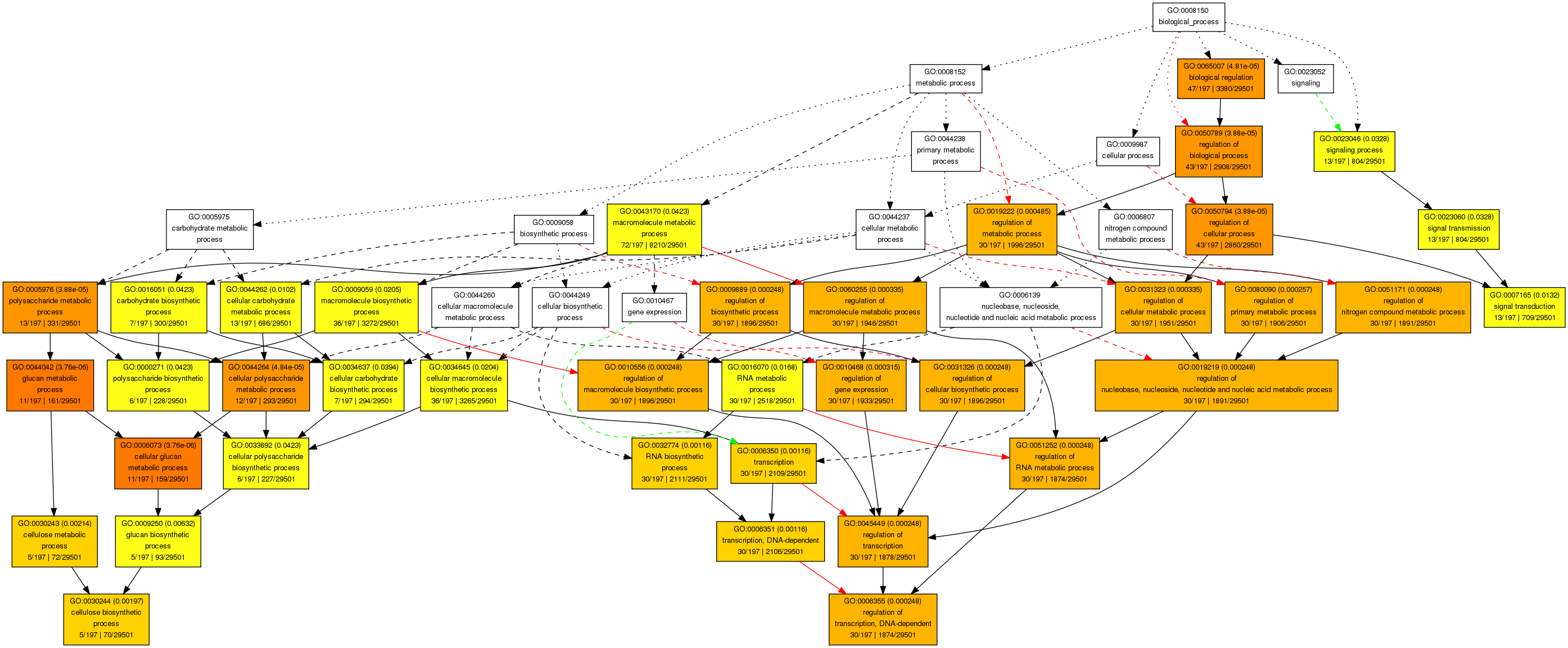

Supplement: Supplementary file 1 [file ijms-21-04316-s001.zip › Supplementary Materials 2020June13/Figure S4.tif]

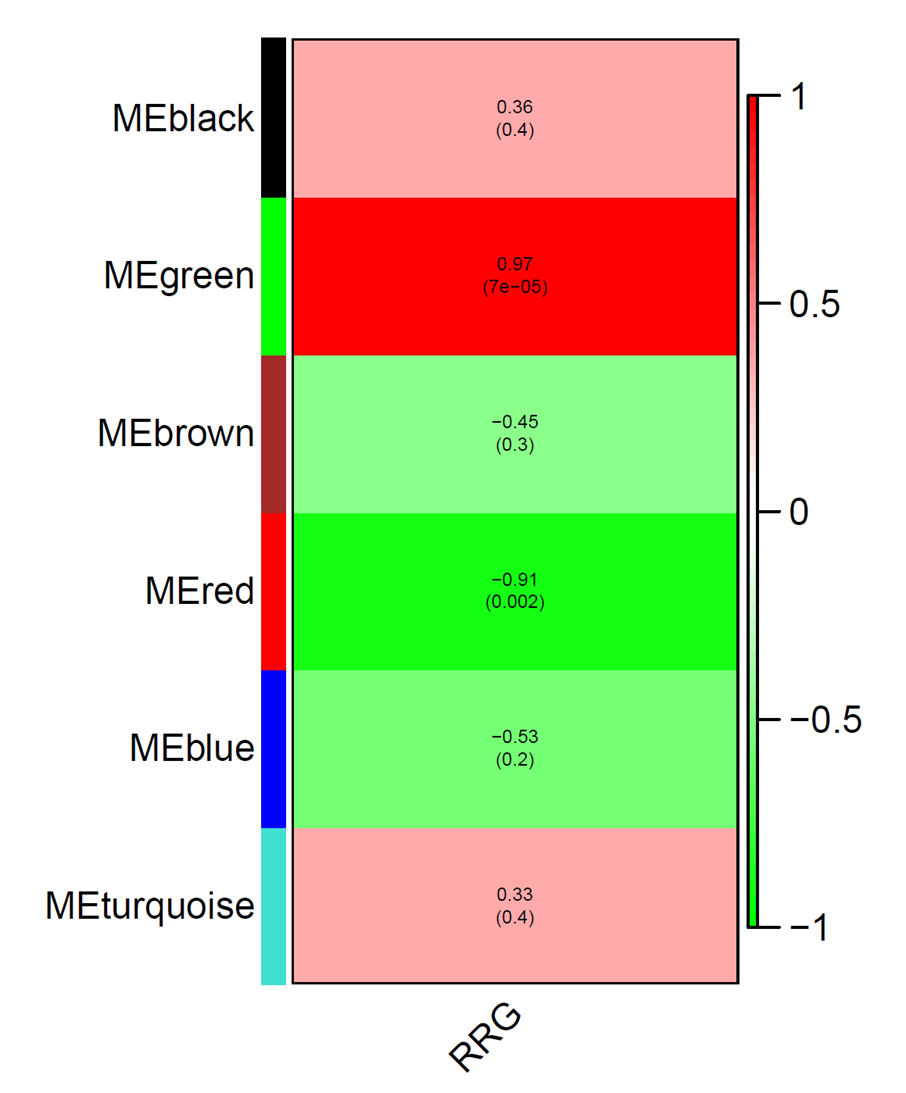

Supplement: Supplementary file 1 [file ijms-21-04316-s001.zip › Supplementary Materials 2020June13/Figure S5.tif]
